# A Study of the Mechanism and Separation of Structurally Similar Phenolic Acids by Commercial Polymeric Ultrafiltration Membranes

**DOI:** 10.3390/membranes12030285

**Published:** 2022-03-01

**Authors:** Qinshi Wang, Yun Zhang, Xianli Zhang, Qi Li, Mingcong Huang, Shasha Huang, Qianlian Wu, Zhishu Tang, Linmei Pan, Yue Zhang, Hongbo Liu, Bo Li, Huaxu Zhu

**Affiliations:** 1 Jiangsu Collaborative Innovation Center of Chinese Medicinal Resources Industrialization, Nanjing University of Chinese Medicine, Nanjing 210023, China; 15850600541@163.com (Q.W.); zycloud1016@163.com (Y.Z.); zhangxli2022@163.com (X.Z.); lq23400@163.com (Q.L.); wongmingcong@163.com (M.H.); ssh_1126@126.com (S.H.); wql961114@163.com (Q.W.); linmeip@126.com (L.P.); zhyue@njucm.edu.cn (Y.Z.); 2 Jiangsu Research Center of Botanical Medicine Refinement Engineering, Nanjing University of Chinese Medicine, Nanjing 210023, China; 3 Simcere Pharmaceutical Co., Ltd., Nanjing 210042, China; 4 Shaanxi Collaborative Innovation Center of Chinese Medicinal Resources Industrialization, Shaanxi University of Chinese Medicine, Xianyang 712046, China; tzs6565@163.com (Z.T.); 15319084280@126.com (H.L.); 5 The First Clinical Medical College, Nanjing University of Chinese Medicine, Nanjing 210023, China

**Keywords:** adsorption, penetration mechanism, phenolic acid compounds, LogD, charge

## Abstract

This study examined the behavior and penetration mechanisms of typical phenolic (benzoic) acids, which determine their observed penetration rates during membrane separation, focusing on the influence of electrostatic and hydrophobic solute/membrane interactions. To understand the effects of hydrophobicity and electrostatic interaction on membrane filtration, the observed penetration of five structurally similar phenolic acids was compared with regenerated cellulose (RC) and polyamide (PA) membranes at different solute concentrations and solution pHs. Variation partitioning analysis (VPA) was performed to calculate the relative contributions of electrostatic and hydrophobic effects. The penetration of phenolic acids was mainly influenced by the electrostatic interaction, with salicylic acid having the highest penetration. Penetration of phenolic acids through the PA membrane decreased from 98% at pH 3.0 to 30–50% at pH 7.4, indicating the dominance of the electrostatic interaction. Moreover, based on its hydrophobicity and greater surface charge, the PA membrane could separate binary mixtures of protocatechuic/salicylic acid and 4-hydroxybenzoic/salicylic acid at pH 9.0, with separation factors of 1.81 and 1.78, respectively. These results provide a greater understanding of solute/membrane interactions and their effect on the penetration of phenolic acids through polymeric ultrafiltration membranes.

## 1. Introduction

Phenolic acids, common secondary metabolites of plants, are found widely in vegetables (e.g., artichokes, olives, and maize), fruits (e.g., grapes, apples, pears, cherries, and berries), beverages, cereals, and other foods [1]. Phenolic acids constitute an important source of bioactive compounds in the human diet because of their wide distribution in plants [2]. In addition, phenolic acids are essential active components of traditional Chinese medicines (TCM) and are found in over 100 TCMs, including Pinellia ternata, Salvia miltiorrhiza Bunge, and Paeonia lactiflora Pallas [3]. Furthermore, the anti-inflammatory [4,5], anticancer [6], antibacterial [7], anti-oxidation, and anti-oxidant [8] activities of phenolic acids are widely used in medical, food, and personal care products. For example, salicylic acid (SA) is the starting material for some synthetic drugs [9,10], such as aspirin and glyburide, and also a raw material or synthetic intermediate for food preservatives [11], dyes [12], and pesticides [13]. The anti-oxidant, anti-inflammatory, and anti-hyperglycemic activities of protocatechuic acid (PCA) have protective or therapeutic benefits in obesity management [14].

Among the various types of phenolic acid, benzoic acid derivatives have attracted the most research interest [15]. Sharing similar metabolic pathways (shikimate and phenylpropanoid pathways) [16], the chemical structures of these substances are all positional isomers of phenolic hydroxyl or methoxy groups, and various structures are usually found in the same plant [5,17]. For example, 4-HA is a common impurity in natural salicylic acid. Although the primary method for producing salicylic acid is chemical synthesis, 4-hydroxybenzoic acid (4-HA) is often present as a by-product [18]. Therefore, different phenolic acids should be separated and purified as pharmaceutical intermediates, pharmaceuticals, and personal care products.

The main methods currently used to separate phenolic acids are solid-phase extraction (SPE) [19], liquid–liquid extraction (LLE) [20], chromatographic methods [21,22,23,24], and other methods, such as capillary electrophoresis [25] and ionic liquid-modified silica gel [26]. These methods are effective for separation, but they have limitations such as being excessively time-consuming, needing high solvent consumption, complicated to control, and producing environmentally damaging wastes [27,28]. These limitations have highlighted the need for an alternative simple, rapid and effective separation method, which does not need organic solvents, such as membrane separation.

The separation of similar compounds by molecularly imprinted membranes has been investigated. A surface imprinted layer, attached to a PVDF membrane, was able to separate salicylic acid from acetyl salicylic acid [29]. Molecularly imprinted membranes were able to separate phenol or methyl salicylate from salicylic acid [30,31] This approach is innovative, but industrial production and application of molecularly imprinted membranes is complex and challenging [32], so it would be useful to increase understanding of the interactions between similar phenolic acids and commercial membranes, with a view to enhancing their separation.

To the best of our knowledge, there are no reports on the separation of similar phenolic acids using commercial polymeric membranes. However, there are some on the separation of phenolic acids from other components. Phenolic acids (vanillic acid, p-coumaric acid, and ferulic acid) were separated from monosaccharides using nanofiltration membranes. Under high pH conditions, the charge on both the membranes and phenolic acids increased, increasing electrostatic repulsion, whereas monosaccharides were electrically neutral [33]. The much higher retention of gallic acid by Biomax5k membranes, compared with acetovanillone and esculetin, was attributed to repulsion by the charged membrane surface and the moderate adsorption capacity of the hydrophobic polyethersulfone membrane [34]. Electrostatic repulsion was the main reason for the high retention of acetic acid by polyamide and polyethersulfone membranes operated at pH 9 [35].

During membrane filtration with tight ultrafiltration and nanofiltration membranes, size exclusion, electrostatic and hydrophobic interactions are the main mechanisms that determine the retention rates of different compounds [36,37], so separation of charged compounds (phenolic acids, negative charge) from uncharged components (monosaccharides, no charge) is efficient. However, separation of compounds with similar electrostatic (both positively, negatively, or uncharged) and hydrophobic properties, but with quantitative differences in physicochemical properties, may be facilitated by a better understanding of the relationship between physicochemical properties and penetration through commercial membranes.

The main objectives of this study were: (1) to reveal the mechanisms of electrostatic and hydrophobic interactions on the penetration of similar compounds during ultrafiltration; (2) to separate similar compounds with commercial membranes. Five phenolic acids (gallic acid (GA), protocatechuic acid (PCA), 4-hydroxybenzoic acid (4-HA), 3-hydroxybenzoic acid (3-HA), and salicylic acid (SA)) with similar structures (benzoic acids with only the number and position of phenolic hydroxyl groups being different) were selected. To maximize electrostatic and hydrophobic interactions with the membranes, two different materials, polyamide (PA) and regenerated cellulose (RC), with the same molecular weight cutoff (MWCO) of 1000 Da, were selected. The observed penetration rates of phenolic acids at different pH was determined, as well as trial separation of phenolic acid mixtures. To the best of our knowledge, this is the first attempt to separate similar phenolic acids in mixtures by ultrafiltration and may provide reliable guidance for separating and purifying similar natural products using membrane technology.

## 2. Materials and Methods

### 2.1. Chemicals

Five phenolic acid compounds (Shyuanye Corporation, Shanghai, China) were selected as model solutes in this study: gallic acid (GA), protocatechuic acid (PCA), 4-hydroxybenzoic acid (4-HA), 3-hydroxybenzoic acid (3-HA), and salicylic acid (SA) were all of analytical grade (Table 1). All compounds were used without purification, and aqueous solutions were prepared with Milli-Q water.

### 2.2. Membrane Characterization

Two commercial membranes with the same molecular weight cutoff (MWCO) of 1000, regenerated cellulose membrane (MILLIPORE Corp, Burlington, MA, USA), thin-film composite poly(piperazine-amide) membranes (TRISEP Corp, Goleta, CA, USA) were used in this investigation. Table 2 summarizes some relevant characteristics of the investigated membranes.

### 2.3. Experimental Procedure

#### 2.3.1. Static Adsorption and Soaking Experiment

In the adsorption experiments, the total surface area of RC and PA membranes was selected to be 83.6 cm^2^. The membrane was placed in a conical flask containing 130 mL of solution, which was phenolic acids dissolved in ultrapure water, and the concentration was 10 mM. At the same time, a conical flask filled with the same solution but without a membrane was used as a control to exclude the effect of water evaporation on the concentration. Magnetic stirrings were applied in the two conical flasks at 300 rpm and 25 °C, and an equilibration time of 2 h was used. Samples were taken and filtered through cellulose acetate filters with 0.22 µm pore size for subsequent measurement of phenolic acids concentrations.

#### 2.3.2. Membrane Filtration Experiments

The RC and PA membranes were tested in a dead-end filtration cell (UFSC40001, Millipore, Burlington, MA, USA), equipped with a magnetic stirring paddle and external pressure supplied by high-purity nitrogen gas. The effective membrane filtration area was 41.8 cm^2^. The temperature was maintained in penetration experiments at 25 °C. A new membrane specimen was used for each selected phenolic acid compound. New membranes were first soaked in Milli-Q water for approximately 24 h to remove the chemicals used for membrane preservation. Before each experiment, all of the membranes were compacted with Milli-Q water at 2 bar and magnetic stirrer speed 300 r·min^−1^ for at least 30 min until there was no further variation in flux.

After membrane pretreatment, the same membranes were used to measure the flux and penetration rates of solutes selected. During the penetration experiments, the feed solutions were made by dissolving the appropriate chemical amount in Milli-Q water to 10 mmol·L^−1^. Besides, the 1 and 5 mmol·L^−1^ feed solutions were also prepared to study the effect of feed concentration, temperature (25 °C), pressure (2 bar), and the magnetic stirrer speed (300 r·min^−1^) was kept constant. Three hundred mL simulated solutions of five kinds of phenolic acids with different concentrations were added to the membrane cup, and the permeation flux was measured. The membrane filtration was carried out until the permeate volume reached 150 mL. The permeate flow was measured from the permeate volume collected for every minute, using an electronic analytical balance. Each test was repeated three times.

The permeate flux was calculated according to the following equation:

(1) $$J_{v}=\frac{\Delta V}{A_{m}\cdot t}\;$$

where ∆*V* is the permeate volume (L) collected at the same interval *t* (h), and *A_m_* (m^2^) is the active area of the membrane [45].

The observed penetration (similar to the concept of the observed sieving coefficient [46], but with percentage conversion) of every phenolic acid compound was calculated as follows [47,48]:

(2) $${\;P}_{obs}\left(\%\right)=\frac{C_{p,av}}{C_{f}}\times 100\;$$

where $\;C_{p,av}\;$ (mol·L^−1^) is the average solute concentration in the permeate, and $\;C_{f}$ (mol·L^−1^) is the solute concentration in the feed.

The adsorbed amounts of phenolic acids in static adsorption experiments were calculated by:

(3) $$Adsorption=\left(C_{0}{-C}_{A}\right)\frac{V}{A_{m}}\;$$

where $C_{0}$ and $C_{A}$ are the concentration (mol·L^−1^) of phenolic acid solutions when membranes are soaked in it at 0, and 2 h, *V* is the adsorption volume (L), and *A_m_*(m^2^) is the active area of the membrane [45].

The separation factor was calculated as follows [33]:

(4) $$The\;separation\;factor\;=\frac{X_{P}{/Y}_{P}}{X_{f}{/Y}_{f}}\;$$

where $X_{P}\;$ and $Y_{P}$ are the concentrations (mol·L^−1^) of two kinds of phenolic acids in the permeate, respectively; $X_{f}$ and $Y_{f}$ are the concentrations (mol·L^−1^) of two kinds of phenolic acids in the feed, respectively.

### 2.4. Membrane Characteristic Analysis

Before testing, all membranes were dried in a vacuum oven at 35 °C for more than 12 h. Contact angle measurement was performed using a standard Contact Angle Goniometer (DSA100SOP, KRUSS, Hamburg, Germany), and more than nine different locations were selected for each sample. Membrane surface zeta potentials were measured by using an electro-kinetic analyzer (Anton Paar, SurPASS, Graz, Austria).

### 2.5. Analytical Methods

The phenolic acids concentrations were determined by an HPLC system (Agilent Technologies, 1260 Series, Santa Clara, CA, USA) equipped with a UV detector. The LC column used was a ZORBAX SB-C18 (Stable Bond Analytical 4.6 × 250 mm, 5 Micron). Operating conditions were as follows: flux 1 mL·min^−1^, temperature 30 °C, pressure 120 bar, wavelength 280 nm. The mobile phase of gallic acid, protocatechuic acid, 4-hydroxybenzoic acid, and 3-hydroxybenzoic acid was a mixture of 100:0.1 water/formic acid (*v*/*v*) (solvent A) and methanol (solvent B). A linear gradient elution for a total run time of 35 min was used as follows: starting from 95% solvent A and 5% solvent B, reduced to 85% solvent A over 10 min, reduced to 75% solvent A over 10 min, to 65% over 10 min and finally isocratic for 5 min. Salicylic acid was chromatographed in isocratic elution in 15 min, and the mobile phase was a mixture of 100:0.4 water/acetic acid (*v*/*v*) (solvent A) and methanol (solvent B). The system was equilibrated between runs for 30 min using the starting mobile phase composition. All samples were filtered using cellulose acetate filters with 0.22 µm pore size and diluted 1:5 with pure water before HPLC analysis.

The external standard method was applied. Analytical interpolation in a standard calibration curve determined the concentration of phenolic acid compounds from experimental peak areas.

### 2.6. Statistical Analysis

Linear regression was mainly completed with Excel (2019). Variation partitioning analysis (VPA) was introduced to quantify the individual and interactive contributions of electrostatic and hydrophobic interactions to the penetration rates of different phenolic acids. The contribution rates of individual and interactive fouling were calculated from the R^2^ values based on variance partitioning analysis (VPA) [49]. The analysis was launched with the “vegan” package in the R Studio 2021.09.1 [50,51,52].

## 3. Results

### 3.1. Effect of Phenolic Acids’ Adsorption on Membrane

**Figure 1:**
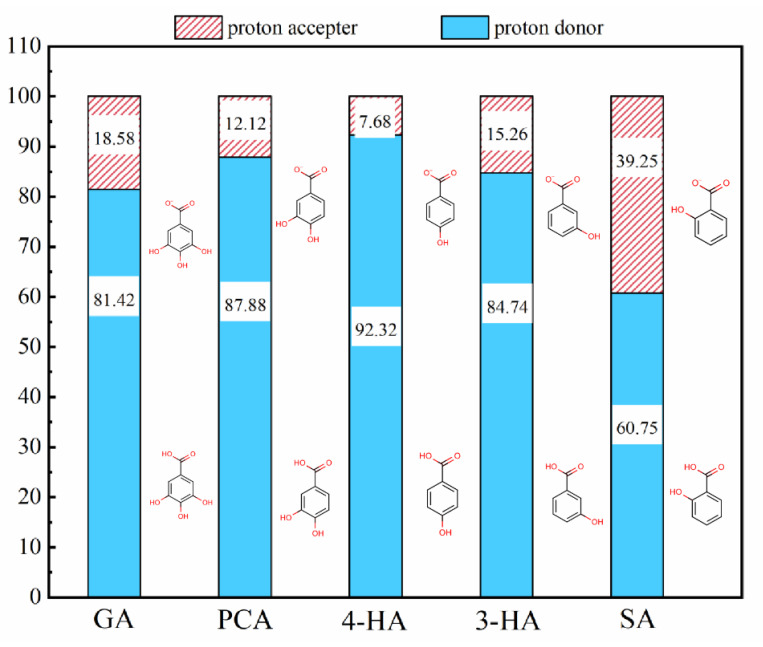
The dissociation percentages of the five phenolic acids at original pH.

**Figure 2:**
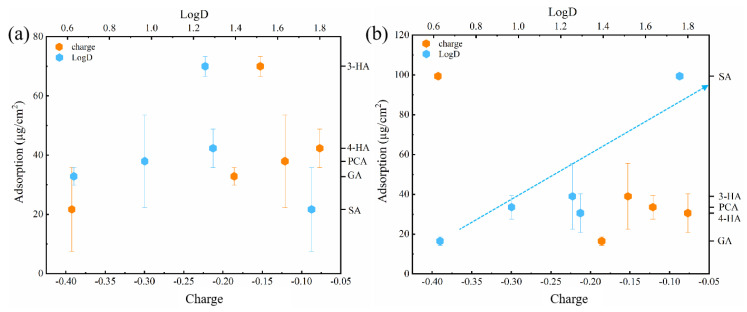
The correlations between amounts of phenolic acids adsorbed onto two membranes ((**a**), for RC membrane; (**b**), for PA membrane) and two important physicochemical properties (charge and the logD values) of phenolic acids. Experimental conditions: feed concentration = 10 mM and 25 ± 2 °C. (The error bar is for adsorption).

Immersion of membranes into phenolic acid solutions resulted in solute adsorption onto the membrane surface, with the amount of each phenolic acid adsorbed differing according to its hydrophobicity and solution charge. At the original pH, the carboxyl group of all five phenolic acids dissociated and had a negative charge in solution (Figure 1). The greater the dissociation, the more negatively charged the compound, SA dissociated the most, with the highest negative charge of −0.39 (Table 1). LogD values reflected the hydrophobicity of compounds. The larger the logD value, the more hydrophobic the compound. Plotting the effects of hydrophobic and electrostatic interactions on adsorption revealed that adsorption onto the two membranes was affected primarily by hydrophobic interactions (Figure 2) [53]; i.e., higher hydrophobicity resulted in increased adsorption. Although SA had the highest logD value (an indicator of hydrophobicity variation with pH), the amount of SA adsorbed to RC membranes was the lowest, apparently because of electrostatic repulsion between SA and the RC membrane. The more hydrophilic surface resulted in lower adsorption of SA onto the RC membrane, relative to the PA membrane.

PCA had greater adsorption than 4-HA onto PA membranes, probably related to the different hydrogen-bonding interactions, based on differences in the numbers of hydrogen-bond donors and acceptors [54,55].

**Figure 3:**
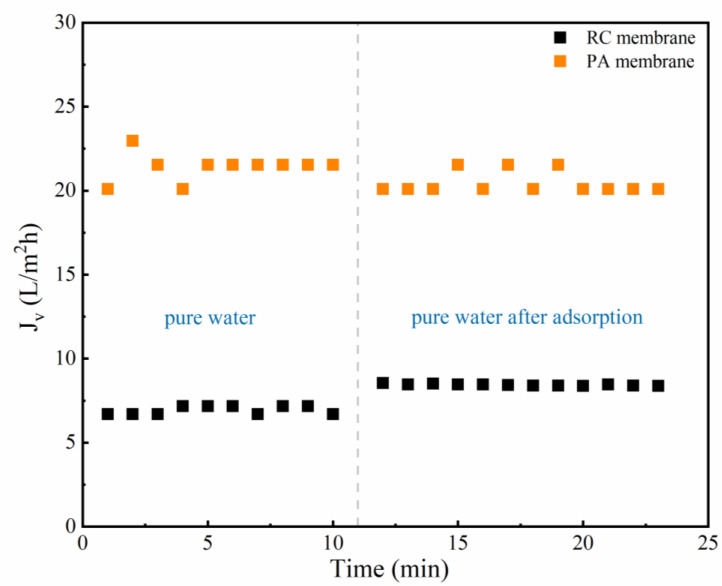
Pure water flux before and after the adsorption of SA on the two membranes. Experimental conditions: 2 bar; feed concentration = 10 mM and 25 ± 2 °C.

The pure water flux after phenolic acid adsorption was measured (Figure 3); the flux of the RC membrane increased after SA adsorption, whereas that of the PA membrane decreased (Figure S1 presented the pure water flux before and after the adsorption of GA, PCA, 4-HA, and 3-HA). This suggests that the change in membrane flux was related to the different properties of the two membranes and the resulting changes following adsorption of the phenolic acids.

**Figure 4:**
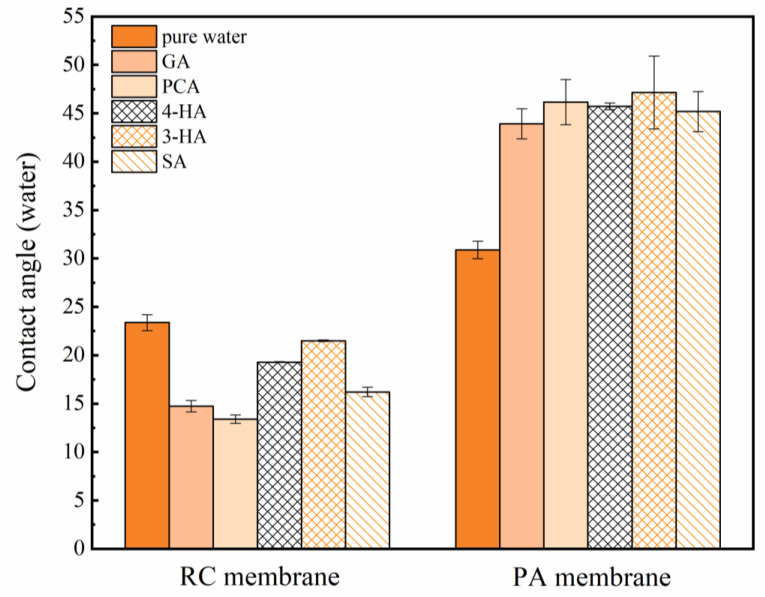
The water contact angle of RC and PA membrane after soaking in pure water and phenolic acids for 2 h. Experimental conditions: feed concentration = 10 mM and 25 ± 2 °C.

**Figure 5:**
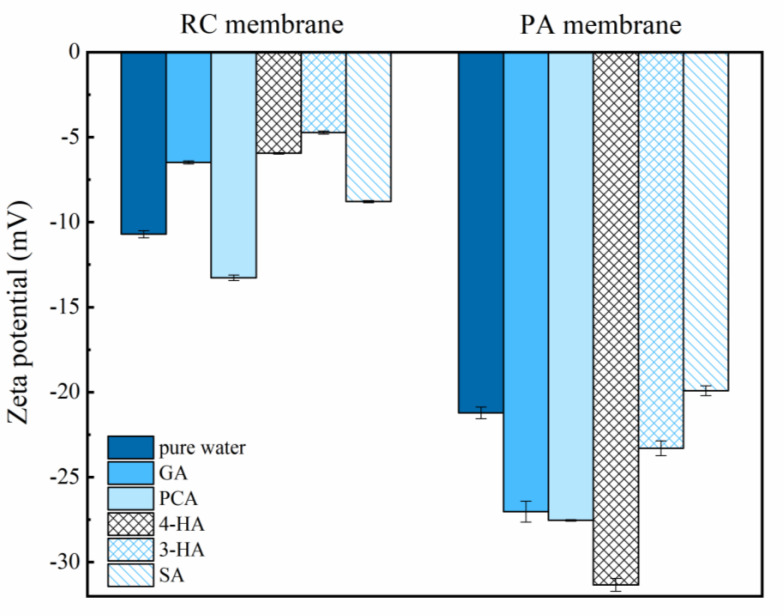
Zeta potential of RC and PA membrane after soaking in pure water and phenolic acids for 2 h. Experimental conditions: feed concentration = 10 mM and 25 ± 2 °C.

Figure 4 and Figure 5 show the contact angle analysis and the zeta potential of the membranes after immersion in the different phenolic acids solutions for two hours. The RC membrane became more hydrophilic and less negative, whereas the PA membrane became more hydrophobic and more negative, after the adsorption of phenolic acids.

### 3.2. Membrane Separation Capability

#### 3.2.1. Effect of Phenolic Acids on Filtration Flux

**Figure 6:**
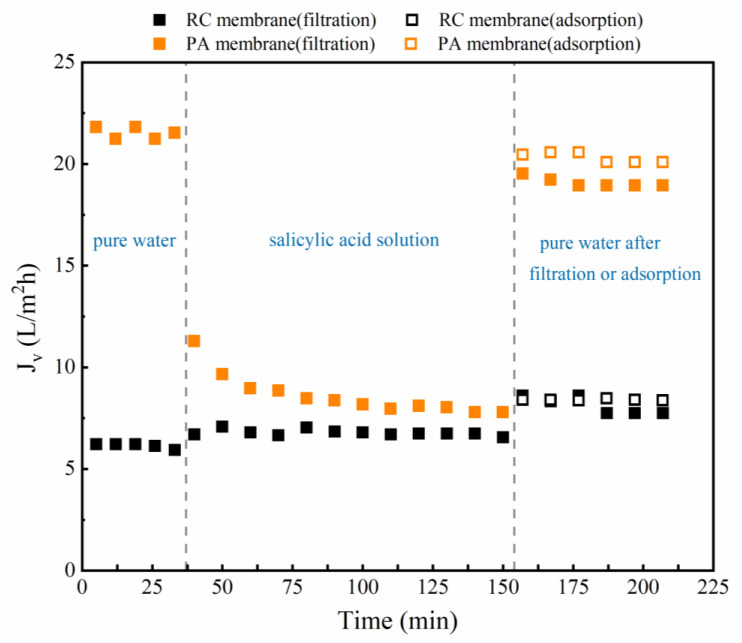
The evolution of the flux of SA during the two membrane filtration processes and water before and after the filtration and adsorption experiments over time. In the last column, the solid point represents the pure water flux after filtration, while the hollow point represents the pure water flux after soaking in phenolic acid solution. Experimental conditions: 2 bar; feed concentration = 10 mM and 25 ± 2 °C.

Permeate flux variations were determined during and after phenolic acid ultrafiltration, using the PA and RC membranes, and compared with phenolic acid adsorption and pure water flux (Figure 6). The permeate flux of the RC membrane remained slightly increased with SA, whereas that of the PA membrane decreased. After filtration and adsorption experiments, the pure water flux increased through the RC membrane and decreased through the PA membrane. Taking into account the difference in membrane molecular weight cut-off (1000 Da) and the molecular weight of the selected phenolic acid (138–179 Da), the flux changes appear to be related to variations in the properties of the membrane surfaces, caused by contact with the solutes (Figure 4 and Figure 5) [56,57] (see Table S1 for the pure water flux loss (%) of different phenolic acids after filtration and adsorption; see Table S2 and Figure S3 for Membrane surface tensions and interfacial free energies analysis; see Figures S5 and S6 for surface SEM morphology of the original and fouled membranes).

#### 3.2.2. Different Phenolic Acids Penetration Rates

The fouling of the PA and RC membranes was relatively light (see above) and the MWCO of both membranes was 1000 Da, much higher than the molecular weight of the tested phenolic acids (there was no significant difference in their Stokes radii), so the membranes cannot retain phenolic acids based on size-exclusion. This suggests that the charge and hydrophobicity of each phenolic acid are the factors determining their penetration rates. The observed penetration rates of the phenolic acids differed between compounds, but were similar for each compound for the two membranes (>90%).

**Figure 7:**
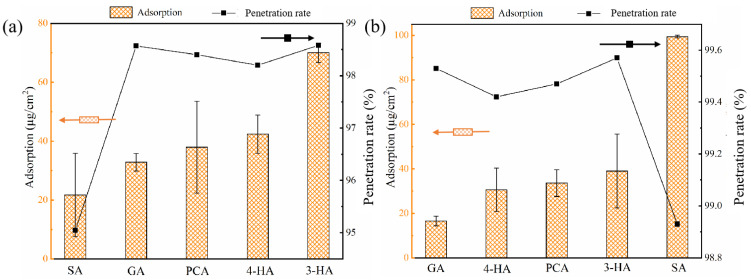
The correlations between amounts of phenolic acids adsorbed onto the two membranes ((**a**), for RC membrane; (**b**), for PA membrane) and the penetration rates. Experimental conditions: feed concentration = 10 mM and 25 ± 2 °C.

The correlations between the amounts of phenolic acids adsorbed on the two membranes and their penetration rates (Figure 7), revealed a trend of decreased penetration rate for the PA membrane with increased solute adsorption (R^2^ = 0.88, Figure S5), in agreement with a previous report [58]. In contrast, no clear penetration trend was observed in relation to adsorption by the RC membrane, which may be related to its greater hydrophilicity.

**Figure 8:**
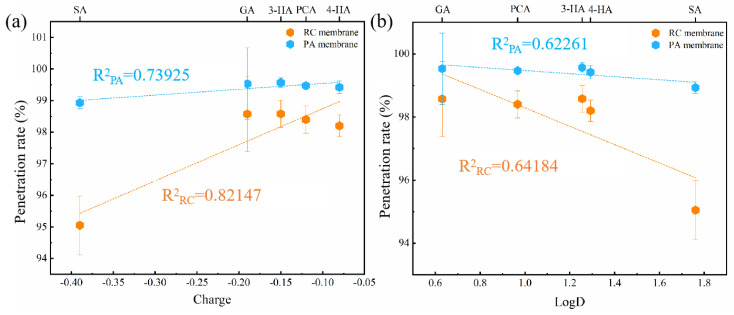
The correlations between the penetration rates of five phenolic acid compounds through the two membranes and two important physicochemical properties ((**a**), for charge, (**b**), for logD values) of phenolic acids. Experimental conditions: 2 bar; feed concentration = 10 mM and 25 ± 2 °C.

Correlations between phenolic acid properties and penetration rates were then analyzed (Figure 8). A better correlation was found between the charge and penetration, i.e., the electrostatic interaction mainly influenced the penetration of phenolic acids through RC and PA membranes. Through both membranes, phenolic acid penetration decreased with their negative charge, because of electrostatic repulsion [59]. In addition, hydrophobic interactions also contributed to the penetration of phenolic acids. Moreover, the larger dipole moment of 3-HA resulted in increased penetration by the RC membrane relative to PCA, in agreement with a previous report [60,61].

#### 3.2.3. Effect of Phenolic Acids’ Concentration

**Figure 9:**
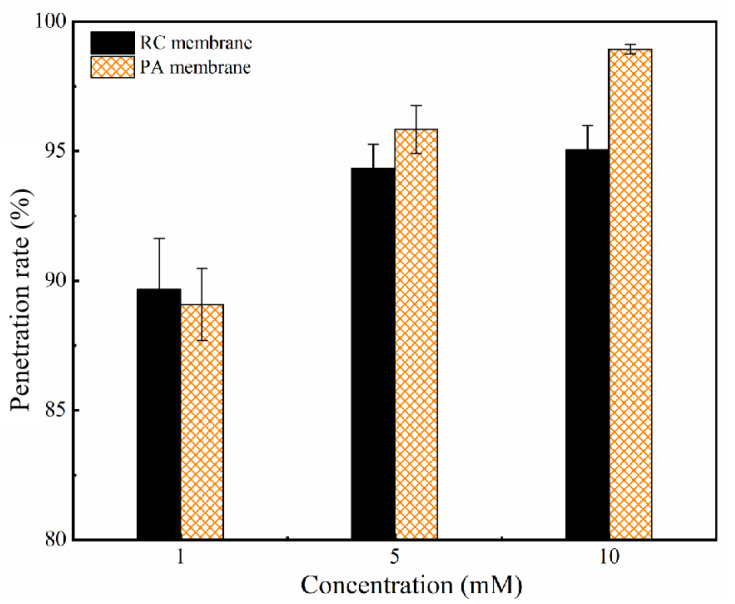
Effect of SA concentration on the penetration rates for these two membranes. Experimental conditions: 2 bar; feed concentration = 1, 5, 10 mM and 25 ± 2 °C.

SA penetration through the RC and PA membranes showed an increasing trend with increasing SA concentration (Figure 9). This may be explained as follows: First, a higher feed concentration may increase solute diffusion and induce a high solute penetrate across the membrane [56,62]. Second, concentration polarization (CP) increased with the solute concentration. The CP layer at high concentration would reduce the water flux and increase solute permeability [63] (Figure S4 present the GA, PCA, 4-HA, and 3-HA’s concentration on the penetration). To reduce the influence of CP and increase the influence of electrostatic and hydrophobic effects, the concentration of phenolic acid in the remaining experiments was limited to 1 mM.

#### 3.2.4. Effect of pH

**Figure 10:**
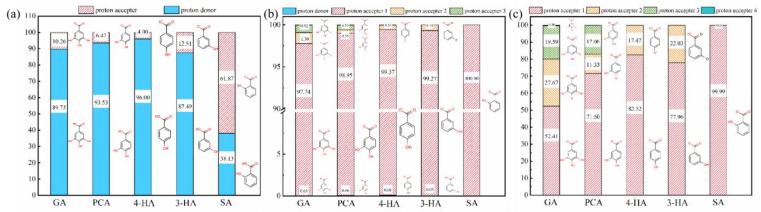
The dissociation percentages of the five phenolic acids at different pH ((**a**), for pH = 3.0; (**b**), for pH = 7.4; (**c**), for pH = 9.0). feed concentration = 1 mM.

The effect of solution pH on the observed penetration rates of the five phenolic acids through the membranes was evaluated. Experiments were performed at pH values of 3.0, 7.4, and 9.0 (except for GA, which degrades irreversibly above pH 7) [64]. The different dissociation behaviors of the phenolic acids at these three pH levels results in different charges (Figure 10). At pH 3.0, the dissociation was basically the same as that of the original solution, except that the carboxyl of salicylic acid dissociated significantly more. At pH 7.4, the carboxyl groups of the phenolic acids in the solution were almost completely dissociated, as well as a small proportion of the hydroxyl groups. At pH 9.0, the increases in negative charge and the decreases in logD values of PCA, 4-HA, and 3-HA resulted from increased hydroxyl dissociation. Only the carboxyl group of SA dissociated, so there was little change in charge, or logD.

**Figure 11:**
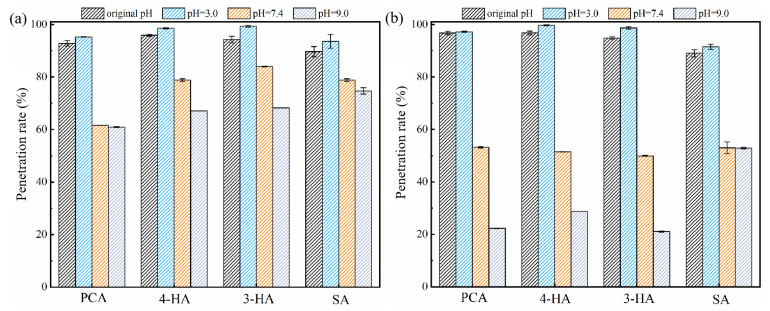
Effect of pH on the penetration rates of phenolic acids for the two membranes ((**a**), for RC membrane; (**b**), for PA membrane). Experimental conditions: 2 bar; feed concentration = 1 mM and 25 ± 2 °C.

Phenolic acid penetration generally decreased significantly with pH (Figure 11 & Table S3). At a higher pH, the membranes become more negatively charged [65] and the phenolic acids more extensively ionized, more negatively charged and more hydrophilic (Table 3), which is in agreement with previous reports [33,34,35,66]. At pH = 9.0, the penetration rate of SA was higher than that of PCA, 4-HA, and 3-HA, Two possible reasons may explain this result. One is that SA has the least charge of the three compounds at pH = 9.0 (−1), the other is that the easier formation of intramolecular hydrogen bonds would make SA less likely to aggregate and its penetration easier [67,68].

Phenolic acid penetration through the PA membrane was influenced by pH more than that through the RC membrane; increasing the pH from 3.0 to 7.4 decreased PCA penetration through the PA membrane from 98% to 30–50% (Figure 11). A more negatively charged and hydrophilic surface, which would have become even more negative and hydrophilic after adsorption of phenolic acids (Figure 4 and Figure 5), may explain why penetration was more variable through the PA membrane.

Previous reports mainly focused on separating phenolic acids from other substances, mostly sugars and phenolics, which are uncharged at pH < 8 [22,61]. However, the phenolic acids selected are all negatively charged at pH above ~2. Although structural differences between them were minor, the effect of pH on their charge and hydrophilicity was different, resulting in different permeabilities.

**Figure 12:**
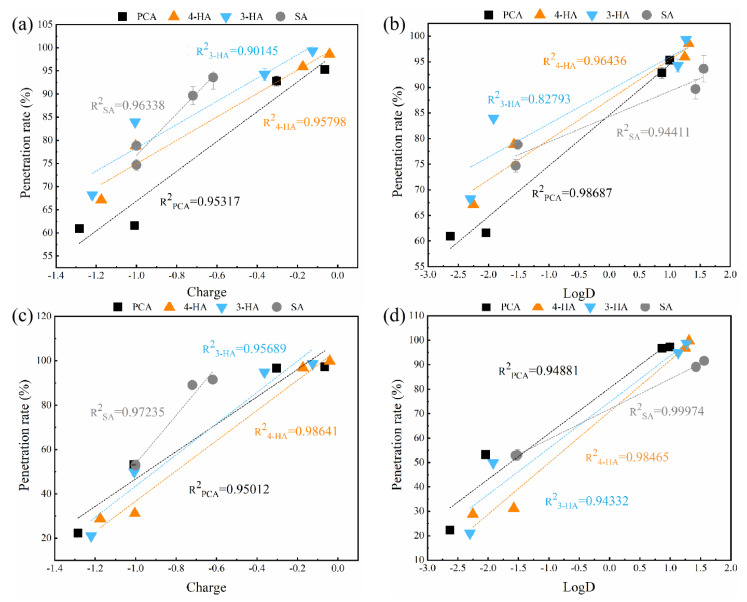
The correlations between the penetration rates of four phenolic acid compounds at different pH on the two membranes ((**a**,**b**) for RC membrane; (**c**,**d**) for PA membrane) and two important physicochemical properties (charge and the logD values) of phenolic acids. Experimental conditions: 2 bar; feed concentration = 1 mM and 25 ± 2 °C.

Plots of penetration against the charge and logD value of the phenolic acids (Figure 12), showed a good correlation between penetration rates and charge, or logD (R^2^ ˃ 0.9). In addition, penetration rates decreased with decreasing logD values, which differed from the observed relationship between adsorption and penetration. Possible reasons are as follows: First, membrane adsorption during filtration initially resulted in the retention of hydrophobic compounds. However, the continued transport of solutes established an equilibrium, resulting in greater retention of relatively hydrophilic compounds than relatively hydrophobic compounds [69,70,71,72]. Second, after reaching equilibrium, electrostatic repulsion would be the dominant mechanism increasing retention of phenolic acids [73].

#### 3.2.5. Relative Influence of Electrostatic and Hydrophobic Interactions on Phenolic Acid Membrane-Penetration

**Figure 13:**
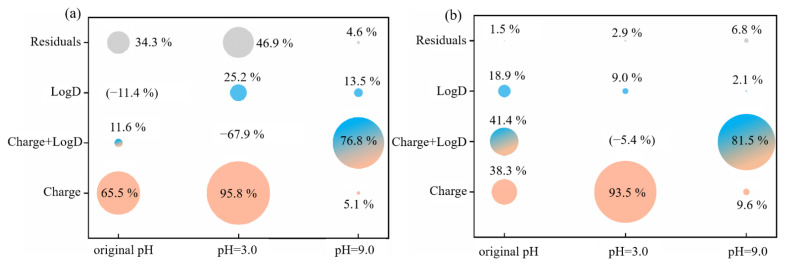
The contributions of different variables to the penetration rates of phenolic acids in two membranes under different solution conditions ((**a**), for RC membrane; (**b**), for PA membrane).

**Figure 14:**
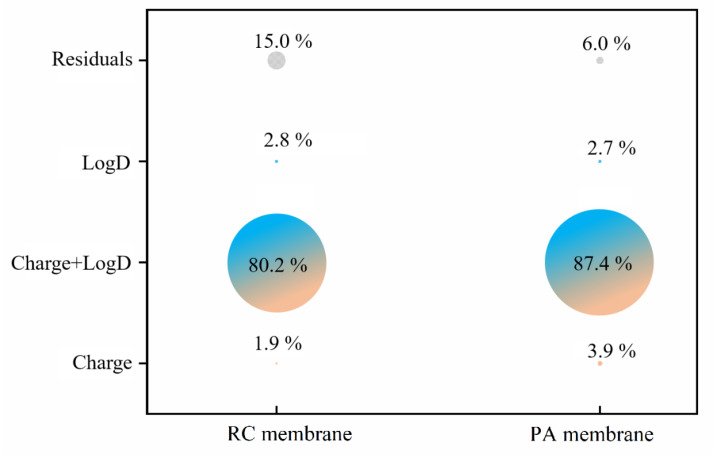
The contributions of different variables to the penetration rates of phenolic acids in RC and PA membrane (overall analysis).

The contribution rate of electrostatic and hydrophobic interactions on phenolic acid penetration through RC and PA membranes, without pH adjustment, at pHs 3.0 and 9.0 was calculated by Variation Partitioning analysis (VPA) (Figure 13 and Figure 14). The result at pH 7.4 is not shown because the charge on all of the phenolic acids was −1.00 at pH 7.4, making calculation impossible.

Without pH adjustment, the contribution of the charge to phenolic acid penetration through the RC membrane was 65.5%. logD (hydrophobic interactions) made a negative contribution (−11.4%) to RC penetration, but it reduced the residual through shared variation of charge (11.6%). However, for the PA membrane, the contributions were charge 38.3%, logD 18.9% and a combined effect of 41.4%. When the solution pH was adjusted to 3.0, charge dominated the effect on both membranes, contributing 95.8% to RC and 93.5% to PA membrane penetration. This provides a plausible explanation for the data in Section 3.2.2 and Section 3.2.4.

VPA of all pH (Figure 14) showed that the combined effect of charge and logD was the primary penetration mechanism for phenolic acids through both RC and PA membranes. In addition, the residuals were 15.0% for RC and 6.0% for PA membranes. Researchers often attributed the effect of pH on solute penetration to electrostatic repulsion between membrane and solutes [33,34,35,66]. However, based on the VPA analysis, the electrostatic interaction does have a major impact, but the hydrophobic effect should never be ignored. In addition, electrostatic and hydrophobic effects had a greater influence during separation with the PA membrane, which had a residual of only 6.0%. This could explain why the surface of the PA membrane was more sensitive to the differences between phenolic acids.

In addition, two QSAR models for RC and PA membranes under all pH conditions showed how the relationships between penetrations and phenolic acid’s parameters were developed, as Equations (5) and (6), respectively. The correlation coefficients of these QSAR models were $R_{RC}^{2}$ = 0.8583 and $R_{PA}^{2}$ = 0.9331. The R^2^ of the QSAR model for PA membrane is closer to 1, and is more significantly influenced by charge and logD.

(5) $$P_{\mathrm{obs}bs\;}=92.33+9.27\;\times \;\mathrm{Charge}+5.50\;\times \;LogD\;$$

(6) $$\;P_{obs}=90.54+29.07\;\times \;\mathrm{Charge}+10.28\;\times \;LogD\;$$

It should be noted that the influence of hydrogen bonding [74,75] on adsorption and penetration was not analyzed. Liu et al. [76] analyzed the impacts of electrostatic and non-electrostatic interactions (including hydrophobic interaction and hydrogen bonding) by comparing adsorption at the isoelectric point (IEP) of different membranes. However, hydrogen bonds are difficult to quantify and usually occur along with hydrophobic interactions. The smaller residual values also verified the dominant contribution of the combined electrostatic/hydrophobic effect and suggest that hydrogen bonding and sieving (steric-hindrance) have little effect on phenolic acid penetration in this study.

Due to the differences in molecular structure and different dissociation states, the penetration rates of different phenolic acids varied significantly with pH and the trends were different (Figure 11). For example, at pH 9.0, penetration of 4-HA, SA and PCA through the PA membrane was 28.8%, 52.9%, and 22.3%, respectively, suggesting that a better separation of similar phenolic acids may be achievable with membrane technology, only by controlling the pH of the mixture solution.

### 3.3. Practical Separation of Different Phenolic Acids in Mixtures

**Figure 15:**
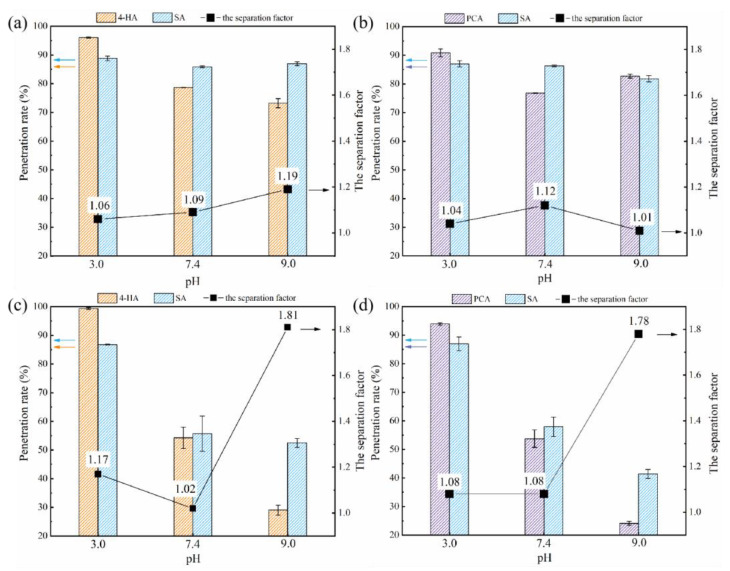
Effect of pH on the penetration rates of 4-HA+SA and PCA+SA for the two membranes ((**a**,**b**), for RC membrane; (**c**,**d**), for PA membrane). The separation factor was calculated by Equation (4).

It appeared from the findings above, that controlling the solution pH could achieve a useful separation of phenolic acids. Two binary mixtures, PCA+SA and 4-HA+SA, were selected to test the effectiveness of pH control. As mentioned above, PCA and 4-HA are often present as impurities in SA. With the PA membrane at pH 9.0, the separation factors of SA/4-HA and SA/PCA were 1.81 and 1.78, respectively. In contrast, the separation factors of the RC membrane were generally small, the penetration rates of PCA, 4-HA, and SA in the mixtures were higher than when they were filtered separately (Figure 15). The explanation could be that adsorption of phenolic acids makes the RC membrane less negatively charged and more hydrophilic, which decreased the electrostatic repulsion and hydrophobic interactions between the membrane surface and all the phenolic acids. In contrast, the PA membrane became more negatively charged and more hydrophobic after contact with phenolic acids, which may be the reason why the PA membrane can separate similar phenolic acids.

The phenolic acids selected are structurally similar, with differences only in the number and position of their phenolic hydroxyl groups. However, quantity differences in charge and hydrophobicity can apparently be used to separate them. The result may provide a facile method for separating phenolic acids and guidance for improving membrane materials [77]. It is well accepted that a more hydrophilic surface would have greater resistance to fouling [78,79]. However, the findings of this study suggest that a more hydrophobic and charged membrane may also be able to separate similar compounds.

## 4. Conclusions

This study analyzed the penetration mechanisms of structurally similar phenolic (i.e., benzoic) acids through polyamide (PA) and regenerated cellulose (RC) ultrafiltration membranes, focusing on the electrostatic and hydrophobic effects. Variation of the feed solution pH significantly affected the observed penetration rates of phenolic acids. Further more, negatively charged, structurally similar phenolic acids can be separated by pH adjustment using commercial ultrafiltration membranes. For example, the penetration of PCA through the PA membrane decreased from 97% at pH 3.0, to 22% at pH 9.0. In addition, the PA membrane produced a superior separation of SA/4-HA and SA/PCA mixtures, with separation factors of 1.81 and 1.78, respectively. These findings facilitate a deeper understanding of the penetration mechanism of some typical phenolic acids, and may provide a facile method for the separation of phenolic acids and guidance for improving membrane materials.

## Figures and Tables

**Table 1:** Physicochemical properties for the selected phenolic acid compounds.

Compound	Molecular Formula	Molecular Structure	Mw (g/mol)	Stokes Radius ^a^ (nm)	PKa ^b^	pH	LogD ^b,d^	Charge ^b,d^	Dipole moment ^c^ (Debye)	H-Bonding Donors ^b^	H-Bonding Acceptors ^b^
GA	C_7_H_6_O_5_	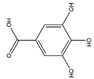	170.12	0.295	3.94	3.27	0.63	−0.19	−0.44	5	5
PCA	C_7_H_6_O_4_	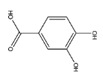	154.12	0.286	4.16	3.27	0.97	−0.12	0.55	4	4
4-HA	C_7_H_6_O_3_	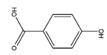	138.12	0.278	4.38	3.29	1.29	−0.08	1.33	3	3
3-HA	C_7_H_6_O_3_	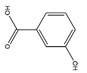	138.12	0.278	3.84	3.10	1.26	−0.15	1.43	3	2
SA	C_7_H_6_O_3_	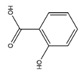	138.12	0.278	2.79	2.61	1.76	−0.39	2.97	3	2

^a^ Data calculated according to the Stokes–Einstein equation based on the assumption of spherical solutes. Moreover, the diffusivity in the Stokes–Einstein equation was obtained from the Wilke–Chang equation [38,39]; ^b^ Data calculated with ChemAxon (http://www.chemicalize.com, accessed on 9 February 2022); ^c^ Data calculated with Chem3D; ^d^ Data determined by 10 mM phenolic acids dissolved in ultrapure water. logD, apparent partitioning coefficient, which took into account the speciation of the compound at various pH levels and was used to reflect the hydrophobicity of compounds [40,41].

**Table 2:** Membrane properties (N.A. = not applicable).

Membrane	PLAC07610	UA60
Abbreviation	RC	PA
Structure	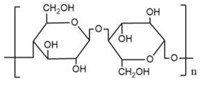	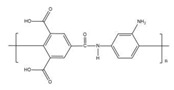
Manufacturer	MILLIPORE	TRISEP
Material	Regenerated Cellulose	Poly (piperazine-amide)
MWCO(Da)	1000	1000
Pore Diameter d ^a^ (nm)	1.59	1.59
Max. pressure (bar)	4.8	7.6 ^b^
Max. temp. (°C)	50	50
pH range	3–13	1–12
NaCl rejection (%)	N.A.	10
Water permeability ^c^ (L/m^2^·h·bar)	2.97	9.19
Contact angle (water) ^c^	23.38	30.88
Zeta-potential (mV) ^d^	−10.71	−21.22

^a^ Calculation using equation [42]. D = 2.20374 × 10^−11^·MW^0.53^; ^b^ Obtained from the literature [43,44]; ^c^ Experimental values measured at 25 °C; ^d^ Experimental values measured at 25 °C, pH = 5.3 ± 0.2.

**Table 3:** The properties of phenolic acids at different pH (feed concentration = 1 mM.).

pH	Charge ^a^				LogD ^a^			
	PCA	4-HA	3-HA	SA	PCA	4-HA	3-HA	SA
original ^b^	−0.22	−0.14	−0.31	−0.72	0.92	1.26	1.17	1.42
3.0	−0.07	−0.04	−0.13	−0.62	0.99	1.31	1.27	1.56
7.4	−1.00	−1.00	−1.00	−1.00	−2.04	−1.58	−1.92	−1.52
9.0	−1.28	−1.18	−1.22	−1.00	−2.63	−2.25	−2.30	−1.55

^a^ Data calculated with ChemAxon (http://www.chemicalize.com, accessed on 9 February 2022); ^b^ The original pH of phenolic acids were 3.62 for PCA, 3.63 for 4-HA, 3.46 for 3-HA, 3.23 for SA.

## Supplementary Materials

The following are available online at https://www.mdpi.com/article/10.3390/membranes12030285/s1, Figure S1: Pure water flux before and after the adsorption of phenolic acids on the two membranes (a for GA, b for PCA, c for 4-HA, d for 3-HA). Experimental conditions: 2 bar, feed concentration =10 mM and 25 ± 2 °C, Figure S2: Effect of SA concentration on the permeate flux (a for RC membrane, b for PA membrane). TMP 2 bar, feed concentration 1, 5, 10 mM, temperature 25 ± 2 °C, Figure S3: The correlations between the interfacial energies for RC and PA membrane and the logD values of different phenolic acids. feed concentration 10 mM, Figure S4: Effect of other phenolic acids concentration on the penetration for these two membranes (a for GA, b for PCA, c for 4-HA, d for 3-HA). Experimental conditions: 2 bar, feed concentration =1, 5, 10 mM and 25 ± 2 °C, Figure S5: The surface SEM images of the original and SA fouled membranes. (a for original RC membrane, b for original PA membrane), Figure S6: The surface SEM images of the original and fouled membranes. (a for GA fouled RC membrane, b for GA fouled PA membrane, c for PCA fouled RC membrane, d for PCA fouled PA membrane, e for 4-HA fouled RC membrane, f for 4-HA fouled PA membrane, g for 3-HA fouled RC membrane, h for 3-HA fouled PA membrane, I for SA fouled RC membrane, J for SA fouled PA membrane), Table S1: The pure water flux loss (%) of different phenolic acids after filtration and adsorption during RC and PA membrane processes, Table S2: RC and PA membranes contact angles, surface tensions and interfacial energies, Table S3: The penetration of phenolic acids (1 mM) at different pH, Table S4: Roughness of membranes. References [80,81,82,83,84,85,86,87,88,89,90,91,92] are cited in the supplementary materials.
