# Supplementary material for: A Study of the Mechanism and Separation of Structurally Similar Phenolic Acids by Commercial Polymeric Ultrafiltration Membranes
Source: Membranes (Basel). 2022 Mar 1;12(3):285. doi: 10.3390/membranes12030285 (PMC8950078; doi:10.3390/membranes12030285)
Supplement: Supplementary file 1 [file membranes-12-00285-s001.zip › membranes-1576642-supplementary.pdf]

SUPPORTING INFORMATION

A study on the mechanism and separation of structurally similar phenolic acids by commercial ultrafiltration membranes

Qinshi Wang [1,2], Yun Zhang [1,2], Xianli Zhang [1,2], Qi Li [1,2], Mingcong Huang [1,2], Shasha Huang [1,3], Qianlian Wu [1,2], Zhishu Tang [4], Linmei Pan [1,2], Yue Zhang [1,2], Hongbo Liu [4], Bo Li [1,2,5,*] and Huaxu Zhu [1,2,*]

[1] Jiangsu Collaborative Innovation Center of Chinese Medicinal Resources Industrialization, Nanjing University of Chinese Medicine, Nanjing 210023, China; 15850600541@163.com (Q.W.); zycloud1016@163.com (Y.Z.); zhangxli2022@163.com (X.Z.); lq23400@163.com (Q.L.); wongmingcong@163.com (M.H.); ssh_1126@126.com (S.H.); wql961114@163.com (Q.W.); linmeip@126.com (L.P.); zhyue@njucm.edu.cn (Y.Z.)
[2] Jiangsu Research Center of Botanical Medicine Refinement Engineering, Nanjing University of Chinese Medicine, Nanjing 210023, China
[3] Simcere Pharmaceutical Co., Ltd., Nanjing 210042, China
[4] Shaanxi Collaborative Innovation Center of Chinese Medicinal Resources Industrialization, Shaanxi University of Chinese Medicine, Xianyang 712046, China; tzs6565@163.com (Z.T.); 15319084280@126.com (H.L.)
[5] The First Clinical Medical College, Nanjing University of Chinese Medicine, Nanjing 210023, China

Submitted to
Membranes
14 January 2022

* Correspond author
E-mail address: Boli@njucm.edu.com (B. Li); Zhuhx@njucm.edu.cn（H. X. Zhu）

1 Flux during different phenolic acids membrane process

1.1 The change of the water flux after soaked in different phenolic acids for 2 h

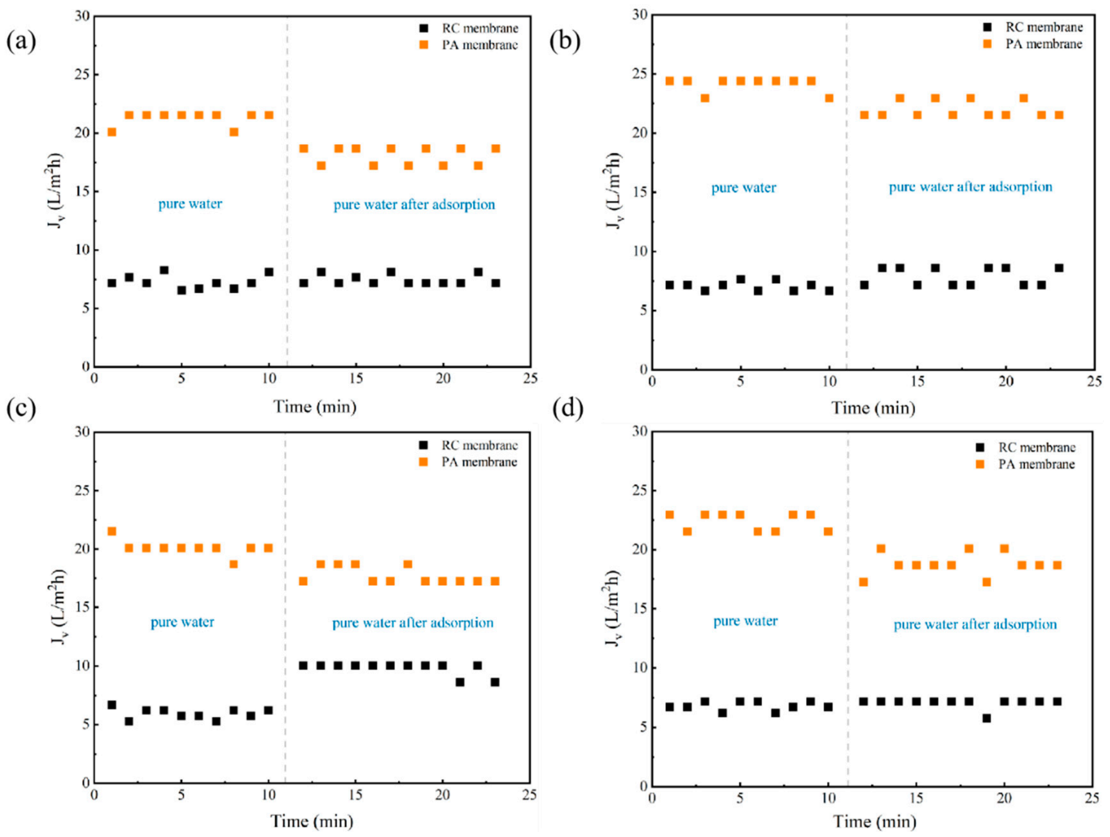

Figure S1. Pure water flux before and after the adsorption of phenolic acids on the two membranes (a for GA, b for PCA, c for 4-HA, d for 3-HA). Experimental conditions: 2 bar, feed concentration =10 mM and 25 ± 2 °C.

1.2 Effect of different phenolic acids on the pure water flux

Table S1

The pure water flux loss (%) of different phenolic acids after filtration and adsorption during RC and PA membrane processes.

Compound	RC membrane		PA membrane	
	filtration	adsorption	filtration	adsorption
GA	-17.74	-6.94	10.39	18.12
PCA	-25.08	-5.37	28.95	23.46
4-HA	-48.92	-53.83	7.41	22.11
3-HA	-20.39	0.69	1.92	-5.71
SA	-33.19	-42.15	9.83	11.52

Figures 2 and S1 shows that the penetration of phenolic acids of the two membranes altered their pure water flux. Table S1 shows the effects of phenolic acids selected on the two membranes specifically. These findings might be explained by membrane fouling or changes in their surface properties. As verified by the adsorption experiment results above and membrane surface tensions and interfacial free energies, which can be seen in supporting information, the change in water flux of these membranes was due to changes in their hydrophobicity following contact with SA rather than membrane fouling.

1.3 Effect of concentration on the permeate flux

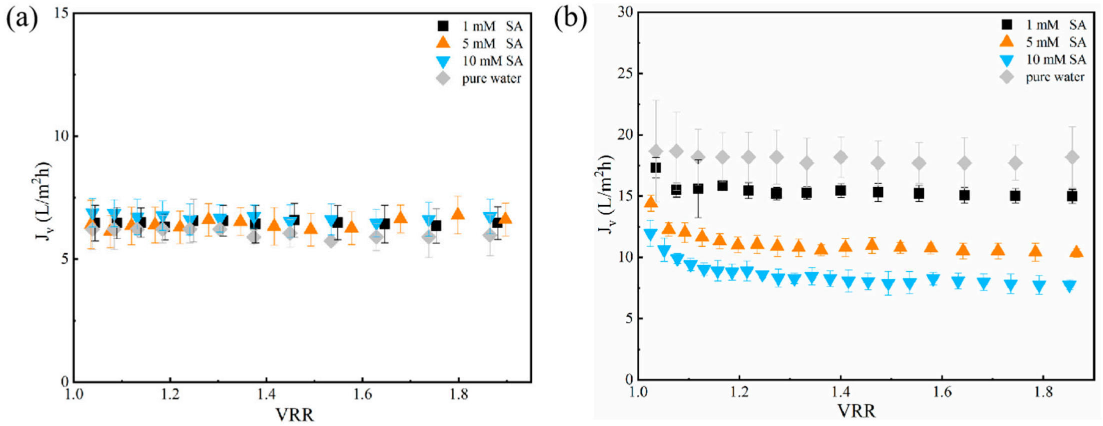

Figure S2. Effect of SA concentration on the permeate flux (a for RC membrane, b for PA membrane). TMP 2 bar, feed concentration 1, 5, 10 mM, temperature 25 ± 2 °C.

We then determined the effect of SA concentration (1–10 mM) on permeate flux in the RC and PA membranes. For the PA membrane, permeate flux showed a decreasing trend along with increased concentration (Figure S2), which agreed with results reported by Cai et al[1]. This could also be explained by CP. At low concentrations, the SA solution showed similar permeation to pure aqueous solution, however, increasing concentration also increased the thickness of the boundary layer as a result of blocked reverse diffusion, with the increased difference in concentration between the boundary layer and bulk phase neutralizing the transmembrane pressure difference and decreasing the permeate flux.

Notably, results for the RC membrane differed, as permeate flux was unaffected by SA concentration (Figure S2). This might be due to the difference in membrane properties. During filtration by the PA membrane, increased SA concentration on the feed side increased solution viscosity, which aggravated the CP effect. An increased CP effect decreased permeate flux for the PA membrane; however, the hydrophilic surface of the RC membrane prevented this from happening.

1.4 Membrane surface tensions and interfacial free energies

Membrane surface tensions and interfacial free energies were derived from contact angles of diiodomethane, glycerol, and a range of phenolic acid solutions, and calculated using Eqs. (S2) and (S3.

Table S2

RC and PA membranes contact angles, surface tensions and interfacial energies.

Membrane	Solution	θ (deg)	γ_m^{LW} (mJ/m²)	γ_m^{+} (mJ/m²)	γ_m^{-} (mJ/m²)	γ_m^{AB} (mJ/m²)	ΔG_{mw} (mJ/m²)	ΔG_{mwm} (mJ/m²)
RC membrane	water	23.38±0.72	37.38	1.95	44.74	18.70	-138.76	19.76
	GA	13.41±0.59	37.38	1.59	52.07	18.18	-142.70	28.67
	PCA	14.73±0.44	37.38	1.62	51.30	18.25	-142.30	27.73
	4-HA	19.29±0.05	37.38	1.78	48.14	18.49	-140.62	23.91
	3-HA	21.50±0.07	37.38	1.87	46.36	18.60	-139.66	21.75
	SA	16.21±0.48	37.38	1.67	50.35	18.33	-141.80	26.59

	water	30.88±0.89	34.87	2.45	34.71	18.44	-130.45	8.68
	GA	45.10±0.41	34.87	3.69	20.44	17.38	-120.21	-9.67
PA	PCA	35.80±0.24	34.87	2.81	29.89	18.32	-127.28	2.58
membrane	4-HA	30.99±0.11	34.87	2.46	34.61	18.44	-130.38	8.55
	3-HA	31.00±0.28	34.87	2.46	34.60	18.44	-130.38	8.54
	SA	37.73±0.06	34.87	2.97	27.94	18.21	-125.93	0.08

Average contact angles of glycerin and diiodomethane were 29.66° and 43.53° for the RC membrane and 28.60° and 44.59° for the PA membrane, respectively.

We then determined changes in membrane-surface tension and interfacial free energy according to the contact angles of diiodomethane, glycerol, and different phenolic acid solutions at the concentration of 10 mM and calculated using Eqs. (S1) and (S2) (Table S2). For deionized water, the RC and PA membranes showed a low surface energy along with mixed electron donor and acceptor polarities. Hydrophilic membranes produce positive ΔG_{mwm} values, whereas hydrophobic membranes exhibit negative ΔG_{mwm} values[2]. Additionally, γ_m^- represents the hydrophilicity of a surface or materials binding to water (γ_w^+)[3,4]. Table S2 shows that the ΔG_{mwm} of the RC and PA membranes was 19.76 and 8.68mJ/m2, respectively. According to γ_m^-, we confirmed that the hydrophilicity of the membranes was RC > PA. Previous studies reported that membrane fouling causes considerable changes in membranes characteristics, including the contact angle, surface charge, and salt retention[5,6]. Bellona et al found that the deposition of trace organic contaminants onto NF270 and thin film composite-S membranes resulted in increased hydrophobicity and decreased surface area[5]. Other studies involving separation of compounds with a lower molecular weight than the MWCO of the membranes, the contact angle was only measured using water regardless of the possible effects caused by the solutes[7-9]. In the present study, we found that the free energy of the RC and PA membranes showed opposite responses following the addition of phenolic acids to the aqueous solution. Compared with the results in water, addition of phenolic acids to the RC membrane resulted in decreased contact angles, increases in the number of electron donors, decreases in the number of electron acceptors, and increased moisture and hydrophilicity. By contrast, under the same conditions, the PA membrane showed the opposite outcomes along with increased hydrophobicity. Moreover, more negative ΔG_{mwm} implied stronger attractive interactions between membrane polymers[2]. Thus, the PA membrane pore shrunk because of strongly attractive forces resulting from the addition of phenolic acids. This might also explain the different change in flux observed in each membrane following contact with the phenolic acids.

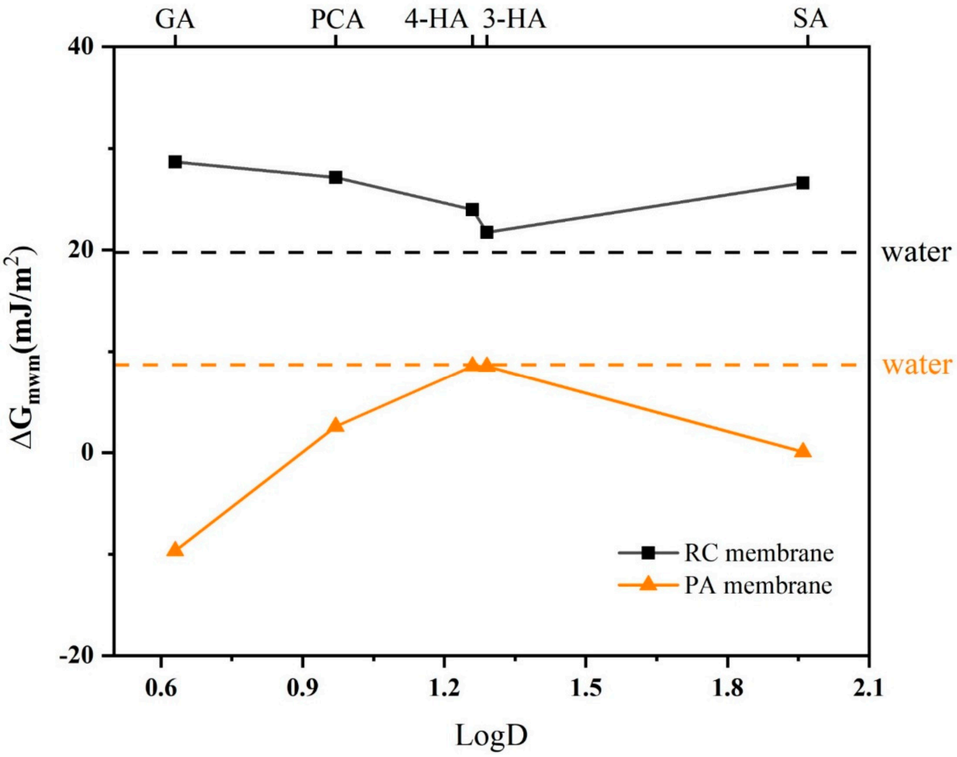

Figure S3. The correlations between the interfacial energies for RC and PA membrane and the logD values of different phenolic acids. feed concentration 10 mM.

We speculated that the hydrophobicity of the RC and PA membranes might be related to the polarity of each phenolic acid. As previously discussed, the RC membrane became more hydrophilic and the PA membrane more hydrophobic following the addition of phenolic acids. logD represents molecule polarity, and generally, a higher logD value indicates a more hydrophobic the compound. The correlation between logD value and membrane hydrophobicity is presented in Figure S2. We found that the ΔG_{mwm} of the PA membrane decreased following contact with each of the five phenolic acids as compared with pure water, whereas the ΔG_{mwm} of the RC membrane increased, indicating the change in hydrophobicity and hydrophilicity, respectively. Additionally, the ΔG_{mwm} of the RC membrane increased along with increasing logD value in all cases, except SA. This might be because the increased adsorbance was a direct result of the higher logD values. However, in the case of SA, its negative charge resulted in a large electrostatic repulsion in the case of the PA membrane and resulted in relatively low adsorption, whereas this effect was less obvious for the hydrophilic RC membrane.

2 Penetration of phenolic acids

2.1 Effect of phenolic acids concentration on the penetration

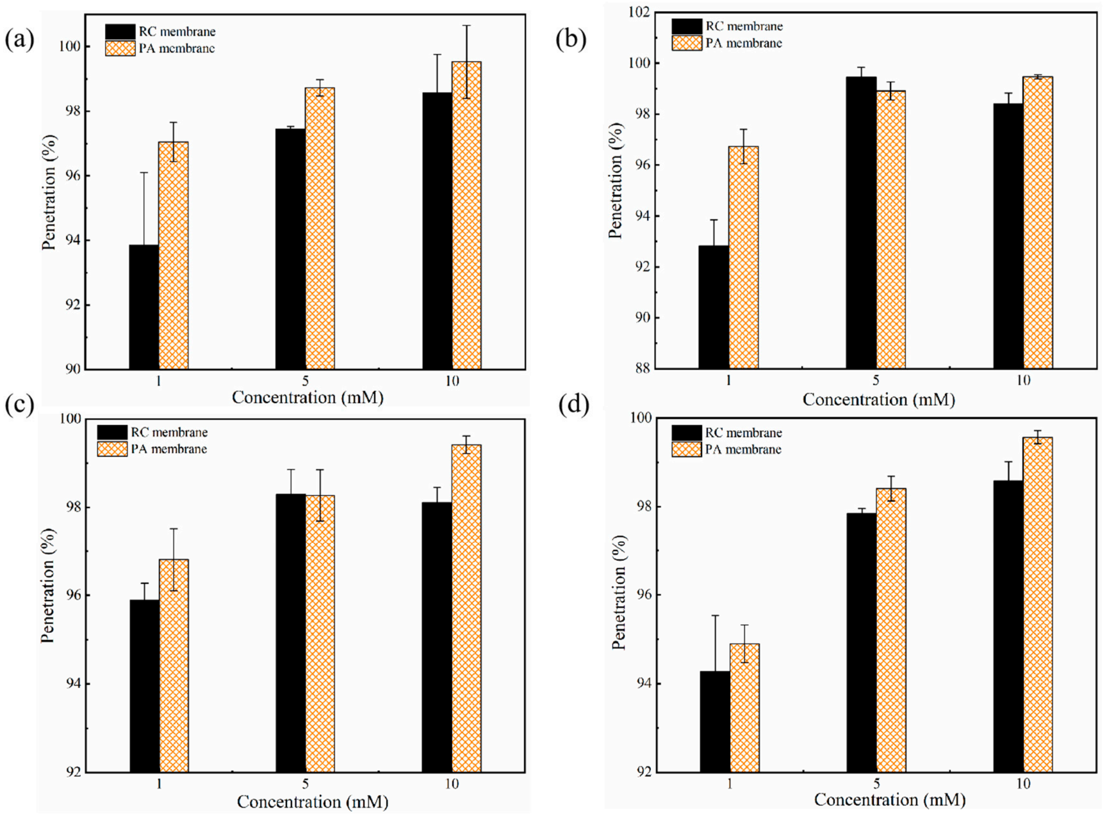

Figure S4. Effect of other phenolic acids concentration on the penetration for these two membranes (a for GA, b for PCA, c for 4-HA, d for 3-HA). Experimental conditions: 2 bar, feed concentration =1, 5, 10 mM and 25 ± 2 °C.

2.2 Effect of pH on the penetration

Table S3

The penetration of phenolic acids(1mM) at different pH.

pH	RC membrane(%)				PA membrane(%)			
	PCA	4-HA	3-HA	SA	PCA	4-HA	3-HA	SA
original	92.83	95.90	94.26	89.66	96.73	96.81	94.90	89.08
3.0	95.27	98.58	99.33	93.61	97.23	99.72	98.71	91.53
7.4	61.60	78.83	83.97	78.82	58.23	31.18	49.94	53.01
9.0	60.93	67.13	68.24	74.69	22.32	28.79	21.04	52.89

3 The surface SEM images of the original and fouled membranes

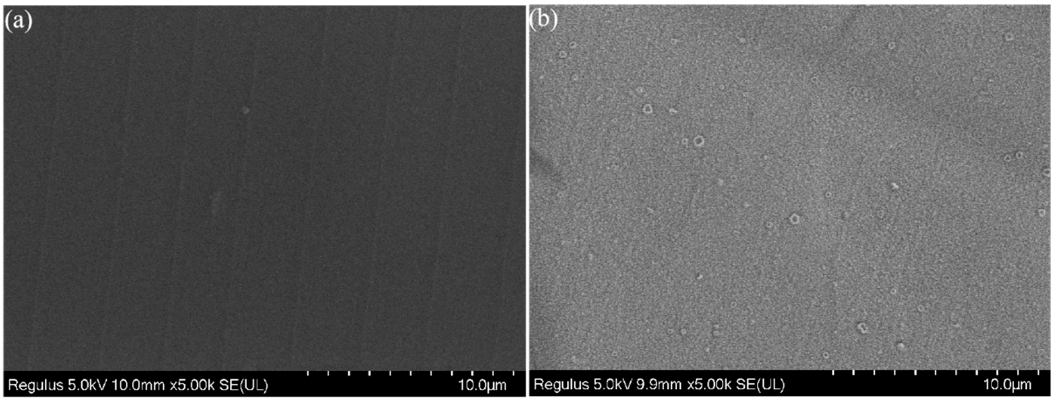

Figure S5. The surface SEM images of the original and SA fouled membranes. (a for original RC membrane, b for original PA membrane).

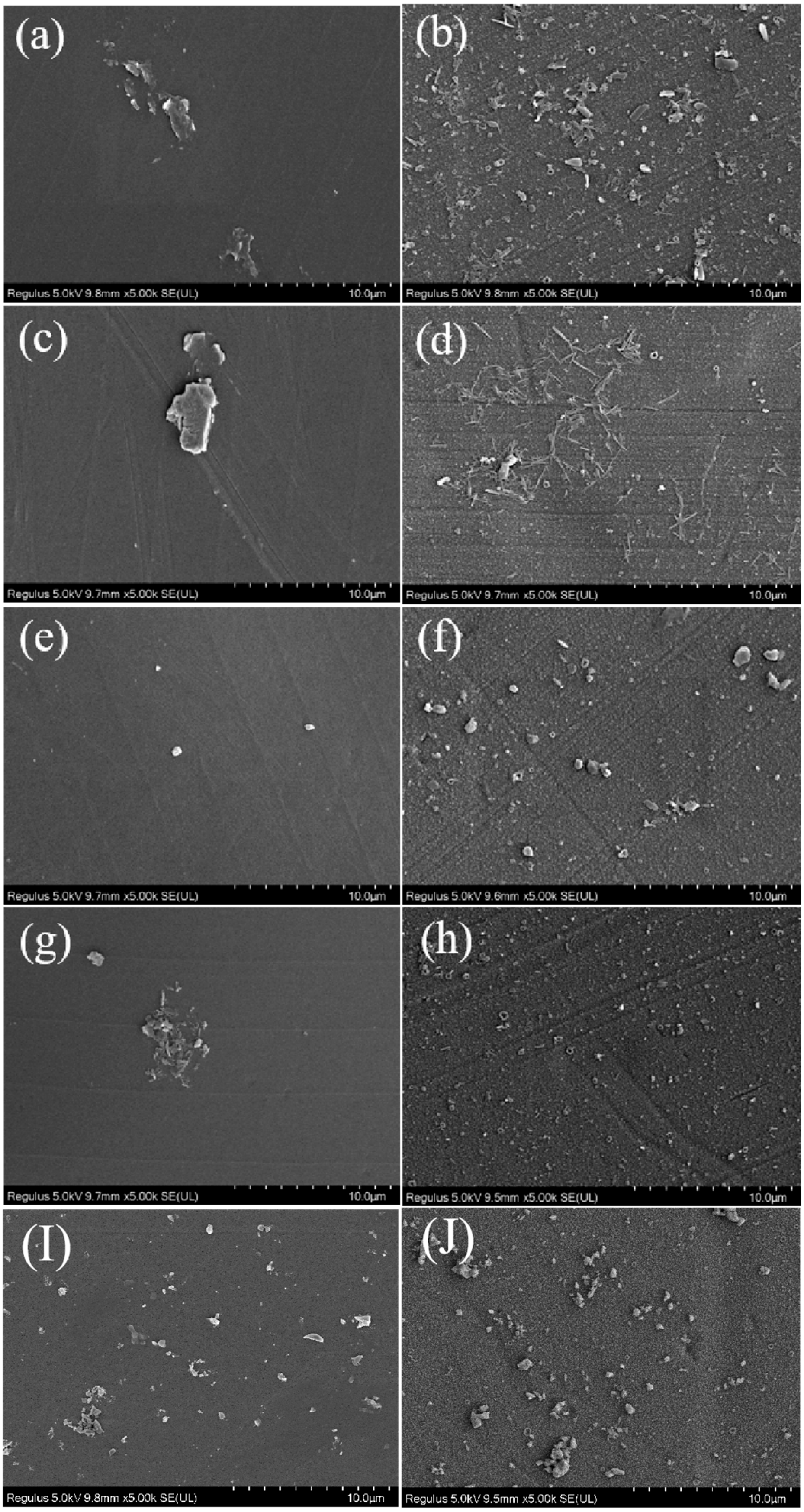

Figure S6. The surface SEM images of the original and fouled membranes. (a for GA fouled RC

membrane, b for GA fouled PA membrane, c for PCA fouled RC membrane, d for PCA fouled PA membrane, e for 4-HA fouled RC membrane, f for 4-HA fouled PA membrane, g for 3-HA fouled RC membrane, h for 3-HA fouled PA membrane, I for SA fouled RC membrane, J for SA fouled PA membrane).

4 Roughness of membranes

Surface tension components were determined from the extended Young equation using a contact angle approach[10,11].

$$\left(1+\frac{\cos\theta}{r}\right)\gamma_l^{TOT} = 2\left(\sqrt{\gamma_s^{LW}\gamma_l^{LW}}+\sqrt{\gamma_s^{+}\gamma_l^{-}}+\sqrt{\gamma_s^{-}\gamma_l^{+}}\right) \quad \text{(S1)}$$

where θ is the contact angle, r is the roughness area ratio. The membrane surface roughness was shown in Table 3, γ^{TOT} is the total surface tension, γ^{LW} is the Lifshitz-van der Waals component, and γ^{+} and γ^{-} are the electron acceptor and electron donor components, respectively. The subscripts *s* and *l* represent the solid surface and the liquid, respectively.

The surface tension parameters of membranes can be determined from equation (7) by measuring the contact angle using two probe liquids with known surface tension parameters.

Table S4

Roughness of membranes.

Membrane	R_a (nm)	R_q (nm)	R_m (nm)	SAD (%)
RC	19.6±6.9	23.1±8.2	101.7±38.6	1.3±0.3
PA	10.9±2.3	15.3±4.0	143.7±35.4	8.4±0.4

R_a: average deviation (above or below) the mean plane. R_q: RMS deviation or z-data standard deviation. R_m: maximum deviation between largest + and - z-values, spread of distribution. SAD: surface area difference, increase in surface area over projected flat plate area (r =1+SAD).

NOTE:

The membrane-water interfacial free energy, ΔG_{mw}, fundamentally describes membrane surface wettability. If the free energy between the identical materials (e.g., the membrane), ΔG_{mwm} indicates the cohesive free energy, and offers a quantitative description of membrane surface hydrophilicity[12,13]. In this paper, ΔG_{mw} and ΔG_{mwm} can be calculated according to[3].

$$\Delta G_{mw} = -2\left(\sqrt{\gamma_m^{LW}\gamma_w^{LW}}+\sqrt{\gamma_m^{+}\gamma_w^{-}}+\sqrt{\gamma_m^{-}\gamma_w^{+}}\right) \quad \text{(S2)}$$

$$\Delta G_{mwm} = -2\left(\sqrt{\gamma_m^{LW}}-\sqrt{\gamma_W^{LW}}\right)^2 - 4\left(\sqrt{\gamma_m^{-}\gamma_m^{+}}+\sqrt{\gamma_w^{-}\gamma_w^{+}}-\sqrt{\gamma_m^{+}\gamma_w^{-}}-\sqrt{\gamma_m^{-}\gamma_w^{+}}\right) \quad \text{(S3)}$$

The subscripts m and w represent the membrane and water, respectively.
Flux loss was obtained from a comparison of the membrane water permeability (J_v) before and after the filtration.

$$FL(Flux\ loss) = \frac{J_i - J_f}{J_i} \quad \text{(S4)}$$

where J_i and J_f are the pure water flux of the new and fouled membranes, respectively.
Volume reduction rate (VRR) was defined as[14]:

$$VRR = \frac{V_0}{V_R} \quad \text{(S5}$$

Where V_0 is initial feed volume and V_R is the volume of the retentate.

References

1. Cai, M.; Hou, W.Z.; Lv, Y.Q.; Sun, P.L. Behavior and rejection mechanisms of fruit juice phenolic compounds in model solution during nanofiltration. *Journal of Food Engineering* **2017**, *195*, 97-104, doi:10.1016/j.jfoodeng.2016.09.024.
2. Wang, J.; Mo, Y.; Mahendra, S.; Hoek, E.M.V. Effects of water chemistry on structure and performance of polyamide composite membranes. *Journal of Membrane Science* **2014**, *452*, 415-425, doi:10.1016/j.memsci.2013.09.022.
3. Oss, C.J. Development and applications of the interfacial tension between water and organic or biological surfaces. *Colloids and Surfaces B: Biointerfaces* **2007**, *54*, 2-9.
4. Liang, S.; Kang, Y.; Tiraferri, A.; Giannelis, E.P.; Huang, X.; Elimelech, M. Highly hydrophilic polyvinylidene fluoride (PVDF) ultrafiltration membranes via postfabrication grafting of surface-tailored silica nanoparticles. *ACS Applied Materials & Interfaces* **2013**, *5*, 6694-6703, doi:10.1021/am401462e.
5. Bellona, C.; Marts, M.; Drewes, J.E. The effect of organic membrane fouling on the properties and rejection characteristics of nanofiltration membranes. *Sep Purif Technol* **2010**, *74*, 44-54, doi:10.1016/j.seppur.2010.05.006.
6. Plakas, K.V.; Karabelas, A.J. A systematic study on triazine retention by fouled with humic substances NF/ULPRO membranes. *Sep Purif Technol* **2011**, *80*, 246-261, doi:10.1016/j.seppur.2011.05.003.
7. Subhi, N.; Verliefde, A.R.D.; Chen, V.; Le-Clech, P. Assessment of physicochemical interactions in hollow fibre ultrafiltration membrane by contact angle analysis. *Journal of Membrane Science* **2012**, *403*, 32-40, doi:10.1016/j.memsci.2012.02.007.
8. Ahmad, A.L.; Yasin, N.H.M.; Derek, C.J.C.; Lim, J.K. Harvesting of microalgal biomass using MF membrane: Kinetic model, CDE model and extended DLVO theory. *Journal of Membrane Science* **2013**, *446*, 341-349, doi:10.1016/j.memsci.2013.07.012.
9. Gao, F.; Wang, J.; Zhang, H.W.; Hang, M.Q.A.; Cui, Z.; Yang, G. Interaction energy and competitive adsorption evaluation of different NOM fractions on aged membrane surfaces. *Journal of Membrane Science* **2017**, *542*, 195-207, doi:10.1016/j.memsci.2017.08.020.
10. Oss, C.J.V. *Interfacial forces in aqueous media*; 1994; pp. 209–210.
11. Wang, J.W.; Mo, Y.B.; Mahendra, S.; Hoek, E.M.V. Effects of water chemistry on structure and performance of polyamide composite membranes. *Journal of Membrane Science* **2014**, *452*, 415-425, doi:10.1016/j.memsci.2013.09.022.

12. Jin, X.; Huang, X.; Hoek, E.M. Role of specific ion interactions in seawater RO membrane fouling by alginic acid. *Environmental science & technology* **2009**, *43*, 3580-3587, doi:10.1021/es8036498.
13. Hurwitz, G.; Guillen, G.R.; Hoek, E.M.V. Probing polyamide membrane surface charge, zeta potential, wettability, and hydrophilicity with contact angle measurements. *Journal of Membrane Science* **2010**, *349*, 349-357, doi:10.1016/j.memsci.2009.11.063.
14. Luo, J.Q.; Ding, L.H.; Wan, Y.H.; Paullier, P.; Jaffrin, M.Y. Application of NF-RDM (nanofiltration rotating disk membrane) module under extreme hydraulic conditions for the treatment of dairy wastewater. *Chem Eng J* **2010**, *163*, 307-316, doi:10.1016/j.cej.2010.08.007.
